# The Domestic Staff—VI

**Published:** 1908-10-03

**Authors:** 


### Nursing Administration.

### THE DOMESTIC STAFF—VI.

### ECONOMY IN NUMBERS.

In a preceding article a few statistics were given to show the cost of service and the average number of maids required in a general hospital. The cost in the twenty-nine hospitals selected for comparison varied from £17 per occupied bed at St. George's Hospital, to £3 6s. at the Leeds General Infirmary. The total of available beds divided among the maids varied in like manner from three at St. Mary's Hospital, at the Royal Free, and at the Glasgow Western Infirmary, to eleven at the Leeds General Infirmary. The more usual figure was from four to six. Excluding the Leeds General Infirmary, which stands for some reason far below the ordinary average, the beds of twenty-eight hospitals divided among the members of the domestic staff show an average of five apiece, and it may be concluded that this is a very fair proportion. Taking a hospital of 150 beds, it gives a domestic staff of thirty persons, including the following departments: Kitchen, ward staff, house staff, nursing home, sewing room, men servants, laundry. On the manner in which this number of servants is distributed throughout the establishment the comfort of all the inmates depends. It is impossible to lay down any hard and fast rules in regard to the numbers in each department, but it is essential that the matron should consider the needs of each section of her household as distinct in itself, and thus introduce order and system into the whole service.

*The Kitchen Staff.*—In hospitals provided, as is now generally the case, with a separate nursing home, it is more convenient from an administrative point of view and possibly even more economical to have a distinct kitchen and staff for the hospital and for the home. It tends to economy, because the strands of the household expenditure are thus sharply defined; but there can be no doubt that such a division entails a certain amount of overlapping in both wages and fuel, not to mention the waste of space. The ideal would be a central kitchen with an annex, both presided over by a well paid and highly trained cook, capable of dealing with the two sections of her household as entirely distinct, and assisted by two head kitchen-maids, one for the nursing home and one for the patients and household. In the moderate-sized hospital, owning but one kitchen, the staff consists of cook and kitchen-maid, with help occasionally in heavy work from one of the men. Nothing is gained, but often much expense incurred, by cutting down the wages of the cook to the level of a general servant. The cook should either be well trained in the institution under a competent person, or pains should be taken to procure a woman who has had systematic instruction not only in cooking, but in institution cooking. There are, unhappily, few opportunities of qualifying for these posts, and little inclination to pay the rate of wages which would encourage superior women to take up this all-important branch of institution work.

*The Wardmaids.*—In the hospital of a normal type there are commonly five or six principal wards, and a wardmaid will be required for each, in addition to one for the theatre. The ward staff required in a hospital of 150 beds will be about six seniors and four juniors, the latter filling the place of the old-fashioned scrubber. The wardmaid is under the ward sister's direction; but not every ward sister understands the art of teaching her her duties. Hence it is necessary that the housekeeper should keep an eye on the way in which the work is done, and much inconvenience is spared if some pains is taken before the promotion of a junior to be senior wardmaid, to see that she fully understands the elements of such work as she will be called on to perform. Where the domestic staff is properly organised there is a definite standard of work, and everything is done exactly in the same way, and done well, whether it be the lighting of a fire with a given number of sticks, or the scrubbing down of a ward.

*The House Staff.*—One head housemaid will probably suffice for the hospital as distinguished from the patients' quarters, and she will require from two to four juniors under her, according to the construction of the building. She will lay the table and wait at meals served in the hospital for members of the staff, and be responsible for the fires, the lighting arrangements, and general cleanliness of the establishment outside the wards.

*The Nursing Home.*—The nursing staff in a hospital such as we have described would number from fifty to sixty. The number of dormitory and other maids required will not be less than two senior and three or four juniors. This is exclusive of the kitchen staff. It is a great mistake to pinch numbers to such an extent that meals are unpunctually and badly served, and every maid is expected to be in two places at once. There are many institutions in which the comfort and leisure of all the nurses is sacrificed for want of an additional maid in the nurses' home.

*Sewing Room.*—One good needlewoman is required here, with an apprentice, and perhaps two, according to the amount of work undertaken.

*Men servants.*—A porter and an engineer are usually all that are required; but the porter must have a junior to relieve him when off duty, and this underling can be turned to good account in many other directions. In hospitals with medical schools the number of men required rises considerably, but with these we are not here concerned.

*Laundry.*—A head laundry-maid and two or three assistants are needed, and a boy is a useful addition to the staff.

The numbers thus reckoned, on a basis of 150 available beds, works out at about thirty. There are many circumstances which tend to raise the number of servants employed, and quite legitimately. But when the number rises well above the average we have shown in many instances to be sufficient, an average which gives a proportion of five beds to each servant, the authorities ought to understand in what direction the excess occurs, and take note whether they are getting good value for the money expended.

